# Involvement of calmodulin and calmodulin-like proteins in plant responses to abiotic stresses

**DOI:** 10.3389/fpls.2015.00600

**Published:** 2015-08-11

**Authors:** Houqing Zeng, Luqin Xu, Amarjeet Singh, Huizhong Wang, Liqun Du, B. W. Poovaiah

**Affiliations:** ^1^College of Life and Environmental Sciences, Hangzhou Normal UniversityHangzhou, China; ^2^Laboratory of Molecular Plant Science, Department of Horticulture, Washington State University, PullmanWA, USA

**Keywords:** calcium signal, calmodulin, calmodulin-like protein, calmodulin-binding protein, signal transduction, abiotic stress

## Abstract

Transient changes in intracellular Ca^2+^ concentration have been well recognized to act as cell signals coupling various environmental stimuli to appropriate physiological responses with accuracy and specificity in plants. Calmodulin (CaM) and calmodulin-like proteins (CMLs) are major Ca^2+^ sensors, playing critical roles in interpreting encrypted Ca^2+^ signals. Ca^2+^-loaded CaM/CMLs interact and regulate a broad spectrum of target proteins such as channels/pumps/antiporters for various ions, transcription factors, protein kinases, protein phosphatases, metabolic enzymes, and proteins with unknown biochemical functions. Many of the target proteins of CaM/CMLs directly or indirectly regulate plant responses to environmental stresses. Basic information about stimulus-induced Ca^2+^ signal and overview of Ca^2+^ signal perception and transduction are briefly discussed in the beginning of this review. How CaM/CMLs are involved in regulating plant responses to abiotic stresses are emphasized in this review. Exciting progress has been made in the past several years, such as the elucidation of Ca^2+^/CaM-mediated regulation of AtSR1/CAMTA3 and plant responses to chilling and freezing stresses, Ca^2+^/CaM-mediated regulation of CAT3, MAPK8 and MKP1 in homeostasis control of reactive oxygen species signals, discovery of CaM7 as a DNA-binding transcription factor regulating plant response to light signals. However, many key questions in Ca^2+^/CaM-mediated signaling warrant further investigation. Ca^2+^/CaM-mediated regulation of most of the known target proteins is presumed based on their interaction. The downstream targets of CMLs are mostly unknown, and how specificity of Ca^2+^ signaling could be realized through the actions of CaM/CMLs and their target proteins is largely unknown. Future breakthroughs in Ca^2+^/CaM-mediated signaling will not only improve our understanding of how plants respond to environmental stresses, but also provide the knowledge base to improve stress-tolerance of crops.

## Introduction

As sessile organisms, plants encounter various types of environmental stresses, which are generally classified into biotic stresses such as insect and pathogen attacks, and abiotic stresses such as unfavorable temperature, lack of or excessive amounts of water, salinity, heavy metal toxicity, chemical toxicity, and nutrient deficiency. On the other hand, the process of industrialization inevitably brings many detrimental effects to the environment. Poorly controlled release of wastes from industrial processes and human life not only adds various toxic chemicals to our water and soil but also release harmful gasses into the atmosphere. Obviously, human activities are creating environmental challenges, making sustained crop production difficult. Classic agricultural technologies such as irrigation, applications of fertilizer, insecticides, fungicides, and chemical phytoprotectants have helped to improve crop yield, but the effects are limited, the costs are high and the impacts on the ecosystems and human health are undesirable and dangerous. Understanding how plants perceive and respond to various environmental stresses provides the necessary platform to create crop varieties which could fit better into the challenging environments, and has become one of the most important tasks for plant scientists around the world.

Calcium is one of the most abundant elements on earth. Ca^2+^ concentration outside the plasma membrane is usually at millimolar level. Since Ca^2+^ can form insoluble compounds with phosphate derivatives and complex with macromolecules, high levels of cytosolic Ca^2+^ are toxic to cells. Ca^2+^ concentration in the cytoplasm and nucleus is usually maintained at 50–100 nM under resting conditions (Reddy, 2001; Yang and Poovaiah, 2003). Ca^2+^ gradient across the plasma membrane as well as inner membrane system are involved in cell signaling process controlled by stimulus responsive Ca^2+^ permeable channels, Ca^2+^ pumps and Ca^2+^/H^+^ exchangers (Reddy, 2001; Kudla et al., 2010). Accumulating evidence reveals that various external stimuli such as gravity, light, cold, heat, drought, water-logging (hypoxia), salt, wind, touch, wounding, and pathogen attacks can quickly induce elevations in cytosolic Ca^2+^ concentration (Poovaiah and Reddy, 1993; Evans et al., 2001; Reddy, 2001; Snedden and Fromm, 2001; Zhu, 2001). Signal-induced nuclear Ca^2+^ changes have also been documented (Van Der Luit et al., 1999; Pauly et al., 2000), but they are not as well studied as cytosolic Ca^2+^ transients (Reddy et al., 2011). The excessive amount of Ca^2+^ in cytoplasm is quickly moved out of the cell or pumped back into the endogenous Ca^2+^ reservoirs such as vacuole and endoplasmic reticulum (ER) through the involvement of Ca^2+^ pumps and Ca^2+^/H^+^ exchangers distributed on the plasma membrane and inner membrane system (Reddy, 2001; Kudla et al., 2010). Interestingly, the transient changes of intracellular Ca^2+^ concentration triggered by various stimuli differ from each other in terms of amplitude, duration, frequency, and spatial distribution inside the cell; and these stimulus-specific Ca^2+^ transients are named calcium signatures by Webb et al. (1996). Stimulus-specific signals are decoded by downstream effector proteins to generate specific or overlapping responses (Poovaiah et al., 2013). These effectors include Ca^2+^ sensor proteins which are represented by three major types in plants, namely calmodulin (CaM)/CaM-like (CML) proteins, calcium-dependent protein kinases (CDPKs) and calcineurin B like (CBL) proteins (Luan, 2009). In this review, our primary focus will be limited to CaM/CMLs and their important roles in plant abiotic stress signaling and responses.

## CaMs AND CMLs

Calmodulin is a ubiquitous Ca^2+^-binding protein which exists in all eukaryotes (Snedden and Fromm, 1998; Yang and Poovaiah, 2003; McCormack et al., 2005; Kim et al., 2009; Du et al., 2011). It is a small acidic protein composed of two pairs of EF-hands located at both the N- and C-terminus. In *Arabidopsis*, seven genes encode four CaM isoforms (CaM1/4; CaM2/3/5; CaM6; CaM7), which differ only in one to five amino acid residues (McCormack and Braam, 2003; McCormack et al., 2005). It has been reported that different CaM isoforms differ in binding and regulating downstream effectors (Lee et al., 2000; Yoo et al., 2005). The slight differences in their structural features may have considerable impacts on their binding to targets (Yamniuk and Vogel, 2005).

In addition to canonical CaM, there are 50 genes coding for CaM-like proteins in the *Arabidopsis* genome which are made of varying number of EF hands and share at least 16% of overall sequence identity with canonical CaM (McCormack and Braam, 2003). Similarly, five *CaM* and 32 *CML* genes, respectively are reported in the rice genome (Boonburapong and Buaboocha, 2007). Despite having four EF hands, most CMLs show low (less than 50%) overall similarity to CaMs (McCormack and Braam, 2003; Boonburapong and Buaboocha, 2007; Perochon et al., 2011). Several *Arabidopsis* CMLs, including CML37, 38, 39, and 42 displayed an electrophoretic mobility shift in the presence of Ca^2+^, indicating that, like CaMs, CMLs also act as Ca^2+^ sensors (Vanderbeld and Snedden, 2007; Dobney et al., 2009). Besides EF-hands, CaMs and CMLs do not carry any known functional domain, and hence usually have no enzymatic or biochemical functions. So far the only exception is CaM7 from *Arabidopsis* which was reported to specifically bind Z-/G-box in a Ca^2+^-dependent manner and act as a transcription factor to regulate light-responsive gene expression and light morphogenesis (Kushwaha et al., 2008). Therefore, identifying CaM/CML targets and understanding the impacts of CaM/CML-binding on their functional behaviors are the major challenges in deciphering the functional significance of CaM/CMLs at molecular, biochemical, and physiological levels.

It is well-documented that Ca^2+^-binding-induced conforma tional changes in CaMs and CMLs usually increase their binding affinity to downstream targets through hydrophobic and electrostatic interactions (Snedden and Fromm, 1998; Hoeflich and Ikura, 2002). A stretch of 16–35 amino acids in the target proteins called CaM-binding domain (CaMBD) is usually necessary and sufficient for its interaction with CaM (Rhoads and Friedberg, 1997; Hoeflich and Ikura, 2002). In some cases, CaM interacts with its target proteins in a Ca^2+^-independent manner, and this kind of interaction requires that the target proteins carry an IQ motif, a stretch of amino acids fitting a conserved pattern of IQXXX(R/K)GXXXR where I could be replaced with “FLV” and “X” represents any amino acid residue (Hoeflich and Ikura, 2002; Yamniuk and Vogel, 2004). CMLs could follow similar models to interact with their targets; however, this assumption requires experimental verification. Usually, CaMBDs are not conserved in their primary structure, however, most of the Ca^2+^-dependent CaMBD peptides share a conserved secondary structure, a basic amphipathic helix with hydrophobic residues arranged on one side and positively charged residues arranged on the other side (Snedden and Fromm, 2001; Du and Poovaiah, 2004). Hence, most CaM and CML target proteins have to be identified empirically.

## Targets of CaMs and CMLs

As mentioned above, the interactions between CaM/CMLs and target proteins are usually Ca^2+^-dependent; regular strategies used for detection of protein–protein interaction including yeast-two-hybrid and coimmunoprecipitation are not effective and fruitful in identifying CaM/CML-binding proteins. The majority of the CaM-binding proteins (CBPs) from plants were identified by screening cDNA expression libraries with labeled CaM as probes (usually ^35^S-labled; Fromm and Chua, 1992; Reddy et al., 1993; Yang and Poovaiah, 2000b). Another effective approach to identify CBPs is utilizing protein microarray (Popescu et al., 2007); however, false positive identification is still a major concern and making protein chips with adequate coverage is currently a challenge. Accumulated results indicated that CaM bind to a variety of CBPs in plants, which include kinases, phosphatases, transcription factors, receptors, metabolic enzymes, ion channels and pumps, and cytoskeletal proteins (Snedden and Fromm, 2001; Bouche et al., 2005; Kim et al., 2009; Du et al., 2011; Reddy et al., 2011). Hence, it is reasonable to conclude that, in most cases, CaMs and CMLs act as multifunctional regulatory proteins, and their functional significance is materialized through the actions of their downstream target proteins. CBPs with well-defined CaM-binding domain, CaM-binding property and involved in plant responses to abiotic stresses are listed in **Table 1**.

**Table 1:** Involvement of calmodulins (CaMs), CaM-like proteins (CMLs), and CaM-binding proteins (CBPs) in plant responses to diverse abiotic stresses.

Protein category	Protein name	Plant species	Stress	Reference
CaM	AtCaM3	*Arabidopsis thaliana*	Heat	Liu et al. (2005), Zhang et al. (2009), Xuan et al. (2010)
	AtCaM7	*A. thaliana*	Heat	Liu et al. (2005)
	OsCaM1-1	*Oryza sativa*	Heat	Wu et al. (2012)
	GmCaM4/5	*Glycine max*	Salt	Park et al. (2004)
	ZmCaM	*Zea mays*	Heat	Gong et al. (1997a,b)
	MBCaM1	*Vigna radiata*	Salt	Botella and Arteca (1994)
	NpCaM1	*Nicotiana plumbaginifolia*	Cold	Van Der Luit et al. (1999)
	TaCaM1-2	*Triticum aestivum*	Heat	Liu et al. (2003)
	RsCaM	*Raphanus sativus*	Heavy metal	Rivetta et al. (1997)
CML	AtCML8	*A. thaliana*	Salt	Park et al. (2010)
	AtCML9	*A. thaliana*	ABA, drought, salt	Magnan et al. (2008)
	AtCML12/TCH3	*A. thaliana*	Heat	Braam (1992)
	AtCML18/CaM15	*A. thaliana*	Salt	Yamaguchi et al. (2005)
	AtCML24/TCH2	*A. thaliana*	Heat, cold, H_2_O_2_, ABA, drought, ion stress	Braam (1992), Polisensky and Braam (1996), Delk et al. (2005)
	AtCML37/38/39	*A. thaliana*	Salt, ABA	Vanderbeld and Snedden (2007)
	AtCML42	*A. thaliana*	Drought, ABA	Vadassery et al. (2012)
	OsMSR2	*O. sativa*	Cold, drought, heat, salt, ABA	Xu et al. (2011)
Protein phosphatase	AtPP7	*A. thaliana*	Heat	Kutuzov et al. (2001), Liu et al. (2007)
Protein kinase	AtCBK3/CRK1	*A. thaliana*	Heat	Wang et al. (2004), Liu et al. (2008)
	AtCRCK1	*A. thaliana*	Cold, salt, ABA, H_2_O_2_	Yang et al. (2004)
	AtCRLK1	*A. thaliana*	Cold	Yang et al. (2010a,b)
	OsCCaMK/DMI3	*O. sativa*	ABA, H_2_O_2_, dehydration, oxidative stress	Shi et al. (2012)
	ZmCCaMK	*Z. mays*	ABA, oxidative stress	Ma et al. (2012)
	TaCCaMK	*Triticum aestivum*	ABA, salt	Yang et al. (2011)
	PsCCaMK	*Pisum sativum*	Cold, salt	Pandey et al. (2002)
	PvNADK	*Phaseolus vulgaris*	Cold	Ruiz et al. (2002)
	NtNADK	*N. tabacum*	Oxidative stress, pathogen,	Harding et al. (1997), Karita et al. (2004)
Transcription factor and co-factor	AtSR1/CAMTA3	*A. thaliana*	Cold	Doherty et al. (2009), Du et al. (2009), Kim et al. (2013)
	AtSR2/CAMTA1	*A. thaliana*	Drought, cold, salt, heat	Doherty et al. (2009), Kim et al. (2013)
	AtSR4/CAMTA2	*A. thaliana*	Cold	Kim et al. (2013)
	SlSR1L	*Solanum lycopersicum*	Drought	Li et al. (2014)
	AtMYB2	*A. thaliana*	Salt, drought	Abe et al. (2003), Yoo et al. (2005)
	AtABF2/AREB1	*A. thaliana*	ABA, drought	Popescu et al. (2007), Yoshida et al. (2010)
	AtCBP60g	*A. thaliana*	ABA, drought	Wan et al. (2012)
	AtGTL1	*A. thaliana*	Dehydration	Yoo et al. (2010)
	AtGT2L	*A. thaliana*	Cold, salt, ABA	Xi et al. (2012)
	PtGTL1	*Populus tremula*	Drought	Weng et al. (2012)
	AtBT1-5	*A. thaliana*	Salt, cold, H_2_O_2_, SA	Du and Poovaiah (2004)
	AtBT2	*A. thaliana*	Cold, ABA, H_2_O_2_	Mandadi et al. (2009)
Ion transportor	AtCNGC1	*A. thaliana*	Heavy metal	Sunkar et al. (2000)
	AtNHX1	*A. thaliana*	Salt	Apse et al. (1999), Yamaguchi et al. (2005)
	AtACA4	*A. thaliana*	Salt	Boursiac et al. (2010)
	SCA1	*Glycine max*	Salt	Chung et al. (2000)
	NtCBP4	*N. tabacum*	Heavy metal	Arazi et al. (1999), Sunkar et al. (2000)
	ZmCAP1	*Z. mays*	Anoxia	Subbaiah and Sachs (2000)
	MCamb1/2	*Physcomitrella patens*	Cold, osmotic stress, ABA	Takezawa and Minami (2004)
Metabolic enzyme	AtCAT3	*A. thaliana*	Oxidative stress	Yang and Poovaiah (2002b)
	ZmSOD	*Z. mays*	Oxidative stress	Gong et al. (1997a)
	OsGAD	*O. sativa*	Anoxia	Aurisano et al. (1995)
	ELP/EcPeroxidase	*Euphorbia characias*	H_2_O_2_	Medda et al. (2003), Mura et al. (2005)
	BiGly-I	*Brassica juncea*	Salt, dehydration, heavy metal	Deswal and Sopory (1999), Veena et al. (1999), Singla-Pareek et al. (2003)
Unclassified	AtCaMBP25	*A. thaliana*	Osmotic stress	Perruc et al. (2004)
	AtIQM1	*A. thaliana*	ABA, dehydration	Zhou et al. (2012)
	ZmHsp70	*Z. mays*	Heat	Sun et al. (2000)
	DgHsp70	*Orchardgrass*	Heat	Cha et al. (2012)
	pTCB48	*N. tabacum*	Heat	Lu et al. (1995)

## Ca^2+^/CAM and ROS Crosstalk in Plant Response to Stresses

Reactive oxygen species (ROS) such as hydrogen peroxide (H_2_O_2_), superoxide anion (O_2_^-^), and hydroxyl radical (⋅OH) are usually produced in various physiological processes and serve as a class of second messengers (Van Breusegem et al., 2001; Apel and Hirt, 2004). While controlled production of ROS is essential to signal appropriate actions to protect plants from various environmental stresses, excessive accumulation of ROS causes damages to plant cells. Oxidative stress is defined as disruption of the cellular redox balance, which could be triggered by a wide range of biotic and abiotic stimuli (Rentel and Knight, 2004). Because of its long half-life and excellent permeability, H_2_O_2_ is broadly accepted as the major form of ROS in plant cells. It is well known that H_2_O_2_ can trigger increases in cytosolic Ca^2+^ by activating the Ca^2+^-permeable channels (Price et al., 1994; Rentel and Knight, 2004). On the other hand, H_2_O_2_ production during oxidative burst is also dependent on continuous Ca^2+^ influx, which activates not only the NADPH oxidase, an EF-hand containing enzyme on the plasma membrane (Xing et al., 1997), but also the CaM-binding NAD kinase (NADK), which supplies NADP cofactor for ROS production through NADPH oxidase (Harding et al., 1997; Karita et al., 2004; Turner et al., 2004).

In addition, early studies from heat-stressed maize seedling suggested that ROS homeostasis and the entire antioxidant system including catalase, superoxide dismutase (SOD) and ascorbate peroxidase, could be regulated by Ca^2+^ influx and intracellular CaM (Gong et al., 1997a). Later plant catalases, a class of H_2_O_2_ scavenger enzymes catalyzing its degradation to water and oxygen was found to bind CaM in a Ca^2+^-dependent manner (Yang and Poovaiah, 2002b). The activity of the *Arabidopsis* CAT3 is stimulated by Ca^2+^/CaM rather than Ca^2+^ or CaM alone, but catalases from other organisms such as *Aspergillus niger*, human and bovine, do not interact with CaM (Yang and Poovaiah, 2002b). A peroxidase from *Euphorbia latex*, was also reported to be a CBP activated by Ca^2+^/CaM (Medda et al., 2003; Mura et al., 2005). Evidence also suggests that another class of ROS-scavenging enzyme SOD could be regulated by CaM in maize, although the specific *SOD* gene has not been cloned (Gong and Li, 1995). The critical role of Ca^2+^/CaM in balancing ROS actions was further supported by the observation that the oxidative damage caused by heat stress in *Arabidopsis* seedlings is exacerbated by pretreatment with CaM inhibitors (Larkindale and Knight, 2002).

In addition to these direct regulations on ROS homeostasis, Ca^2+^/CaM-mediated signaling is also well known to regulate ROS-related signal transduction at various stages. Maize *CAP1* encoding a novel form of CaM-regulated Ca^2+^-ATPase was shown to be induced only during early anoxia, indicating its possible role in oxygen-deprived maize cells (Subbaiah and Sachs, 2000). CaM may also participate indirectly in regulating ROS content through the CaM-regulated -aminobutyrate (GABA) synthesis and the GABA shunt metabolic pathway (Bouche et al., 2003). Recently, it was demonstrated that ZmCCaMK and OsDMI3 (also called OsCCaMK) from maize (*Zea mays*) and rice (*Oryza sativa*), respectively, play a critical role in ABA-induced antioxidant actions (Ma et al., 2012; Shi et al., 2012), suggesting a role for CCaMK in plant oxidative stress response. *Arabidopsis* MPK8 was found to be activated by CaM and activated MPK8 suppresses wound-induced ROS accumulation via transcriptional control of *RbohD* expression, revealing a novel mechanism for CaM-mediated signaling to fine-tune ROS homeostasis under wounding stress (Takahashi et al., 2011). Interestingly, some genes encoding CaM-binding transcription factors (*CAMTAs*) and co-factor (*AtBTs*) are responsive to H_2_O_2_, suggesting that CaM-mediated signaling could directly regulate gene expression in plant responses to oxidative cues (Yang and Poovaiah, 2002a; Du and Poovaiah, 2004; Wang et al., 2015).

## CaM/CML-Mediated Regulation of Abiotic Stress Signaling

### Heat Stress

Prolonged high temperature is usually lethal to all organisms; fluctuations in temperature above optimal level, usually called heat shock (HS), impose major stress affecting plant growth and productivity. Almost all organisms including plants synthesize HS proteins (HSPs), a class of chaperons to assure normal function of various client proteins under adversely high temperature conditions. It was observed long ago that HS induced a quick and strong increase in cytosolic Ca^2+^ in tobacco (Gong et al., 1998). Expression of CaM in the maize coleoptiles was found to be remarkably induced during HS and was affected by Ca^2+^ level, suggesting that Ca^2+^ and CaM may be involved in the acquisition of HS-induced thermotolerance (Gong et al., 1997b). Liu et al. (2003) observed an increase in intracellular Ca^2+^ within one min after wheat was subjected to 37°C HS. Expression of CaM mRNA and protein was both induced by HS in the presence of Ca^2+^, and expression of *HSP26* and *HSP70* was stimulated by exogenous application of Ca^2+^. HS-induced expression of *CaM* was 10 min earlier than that of *HSP*s, and both were suspended by pharmacological reagents which interfere with Ca^2+^ signaling. These results indicate that Ca^2+^ and CaM are directly involved in HS signaling (Liu et al., 2003). The Ca^2+^/CaM signaling system was also proposed to be involved in the induction of *HSP* genes in *Arabidopsis* (Liu et al., 2005). Using molecular and genetic tools, Zhang et al. (2009) found that *Arabidopsis* AtCaM3 was involved in the Ca^2+^/CaM-mediated HS signal transduction pathway. *atcam3* loss-of-function mutant showed a pronounced decrease in thermotolerance after 50 min of incubation at 45°C. The compromised thermotolerance of *atcam3* mutant could be rescued by functional complementation with 35S promoter driven AtCaM3, and overexpression of *AtCaM3* in wild-type (WT) background increased thermotolerance of the transgenic plants. Furthermore, the DNA-binding activity of HS transcription factors and the expression of tested HS genes at both mRNA and protein levels were shown to be down-regulated in *atcam3* null mutant and up-regulated in its overexpressing lines upon HS treatment (Zhang et al., 2009). A role for CaM in HS signaling was also demonstrated in rice (Wu and Jinn, 2012; Wu et al., 2012). HS was reported to induce biphasic cytosolic Ca^2+^ transients, and this signature feature was found to be reflected in the HS-induced expression of *OsCaM1-1.* OsCaM1-1 was observed to localize to the nucleus and overexpression of *OsCaM1-1* in *Arabidopsis* resulted in enhanced thermotolerance which coincided with elevated expression of HS-responsive *AtCBK3, AtPP7, AtHSF*, and *AtHSP* at a non-inducing temperature. Nitric oxide (NO) level in plants was found to be elevated by high temperatures (Gould et al., 2003), and exogenous application of NO donor provides effective protection to plants under heat stress (Uchida et al., 2002; Song et al., 2006). However, for a long time it was unknown how NO is involved in protecting plants from damage by HS. Recently, *Arabidopsis* CaM3 was reported to act as a downstream factor of NO in activation of HS transcription factors, accumulation of HSPs and establishment of thermotolerance (Xuan et al., 2010).

Calmodulin-binding proteins have also been shown to play a crucial role in mediating plant responses to heat stress. *pTCB48* encoding a CBP was isolated by screening a cDNA expression library constructed from tobacco cell cultures subjected to HS, and its expression was strongly induced by HS treatment, suggesting a role in HS response (Lu et al., 1995). Maize cytosolic Hsp70 was identified to bind CaM in the presence of Ca^2+^ and could inhibit the activity of CaM-dependent NADK in a concentration-dependent manner, but its possible function in HS response has not been elucidated (Sun et al., 2000). Recently, DgHsp70, a homolog of cytosolic Hsp70 from orchardgrass (*Dactylis glomerata*) was also found to bind AtCaM2 in the presence of Ca^2+^, and the binding of Ca^2+^/CaM decreased the ATPase and foldase activities of this chaperon protein (Cha et al., 2012). PP7 is a ser/thr protein phosphatase which interacts with CaM in a Ca^2+^-dependent manner (Kutuzov et al., 2001). *Arabidopsis AtPP7* was induced by HS and its knockout mutant is impaired in thermotolerance, while the overexpression of *AtPP7* results in increased thermotolerance and increased expression of *AtHSP70* and *AtHSP101* following HS treatment (Liu et al., 2007). Interestingly, AtPP7 was also found to interact with HS transcription factor AtHSF1 implying that AtPP7 could also regulate the expression of HSP genes via AtHSF1 (Liu et al., 2007). However, the mechanistic detail by which AtPP7 dephosphorylates and regulates downstream substrates such as AtHSF1 is not clear. *Arabidopsis* AtCRK1 (CDPK-related protein kinase 1, also called AtCBK3) was identified as a Ca^2+^-dependent CBP (Wang et al., 2004). Liu et al. (2008) found that AtCBK3 activates HSFs which further regulate HS gene expression by binding to HS elements.

### Cold Stress

Ca^2+^ has been recognized as a vital second messenger coupling cold stress to specific plant responses (Knight et al., 1991; Dodd et al., 2010). Researchers found that cold shock and wind initiate Ca^2+^ transients in both cytosol and nucleus in transgenic tobacco (*Nicotiana plumbaginifolia*) seedlings expressing aequorin, and the expression of *NpCaM-1* is induced by both cold shock and wind but mediated by distinct Ca^2+^ signaling pathways operating predominantly in the cytoplasm or in the nucleus (Van Der Luit et al., 1999). Transgenic *Arabidopsis* plants overexpressing *CaM3* showed decreased levels of *COR* (cold regulated) transcripts suggesting CaM may function as a negative regulator of cold-induced gene expression (Townley and Knight, 2002). Genes encoding CMLs, such as *AtCML24/TCH2* and *OsMSR2* (*O. sativa* Multi-Stress-Responsive gene2, a novel CML gene), were also found to be induced by cold treatment and thus, likely participate in transducing cold-induced Ca^2+^ signals (Polisensky and Braam, 1996; Delk et al., 2005; Xu et al., 2011).

In addition, as downstream effectors of Ca^2+^/CaM-mediated signaling, CBPs are also known to be involved in plant responses to cold stress. Ca^2+^/CaM-regulated receptor-like kinase CRLK1, which is mainly localized in the plasma membrane, was found to be involved in cold tolerance (Yang et al., 2010b). CRLK1 carries two CaM-binding sites in both N- and C-termini with affinities for Ca^2+^/CaM of 25 and 160 nM, respectively (Yang et al., 2010b). *crlk1* knockout mutant plants grow and behave like WT plants under regular conditions, but are more sensitive to chilling and freezing treatments than WT plants (Yang et al., 2010b). In addition, cold response genes such as *CBF1, RD29A, COR15a*, and *KIN1* showed delayed responses to cold in *crlk1*, suggesting a positive role for CRLK1 in regulating cold tolerance. MEKK1, which is a member of the MAP kinase kinase kinase family, was shown to interact with CRLK1 both *in vitro* and *in planta* (Yang et al., 2010a). Knockout mutation of CRLK1 abolished the cold-triggered MAP kinase activities, and altered cold-induced expression of genes involved in MAP kinase signaling (Yang et al., 2010a). Therefore, Ca^2+^/CaM-regulated CRLK1 may modulate cold acclimation through MAP kinase cascade in plants. Other CaM-binding kinases are also suggested to be involved in cold acclimation. The expression of *PsCCaMK* in pea (*Pisum sativa*) roots was found to be up-regulated by low temperature or salinity stress (Pandey et al., 2002), and the activity of the Ca^2+^/CaM-dependent NADK was found to be increased by cold shock (Ruiz et al., 2002).

AtSRs/CAMTAs belong to one of the best characterized classes of CaM-binding transcription factors in plants and animals (Reddy et al., 2000; Yang and Poovaiah, 2000a, 2002a; Bouche et al., 2002; Choi et al., 2005; Du et al., 2011; Reddy et al., 2011). In an attempt to understand transcriptional control of CBF2 (a critical regulator of cold acclimation), Doherty et al. (2009) compared the promoter sequence of three CBFs and found seven conserved DNA motifs CM1 to 7 in their promoters. CM2 is a typical AtSR1/CAMTA3 recognition motif, and importantly, the expression of *CBF2* was found to be positively regulated by AtSR1/CAMTA3. Although *camta3* knockout mutant had no phenotypic change under cold stress, *camta1/camta3* double mutant was found to have reduced freezing tolerance (Doherty et al., 2009). Recently, AtCAMTA1, AtCAMTA2, and AtCAMTA3 were shown to participate in cold tolerance by cooperatively inducing *CBF* genes and repressing SA biosynthesis (Kim et al., 2013). These results filled a long-standing knowledge gap between cold induced Ca^2+^ transients and cold-regulated gene expression.

Several *Arabidopsis* MADS box transcription factors were identified as putative CBPs by a high throughput proteomics approach (Popescu et al., 2007). Expression of some of these MADS box genes, including *AGL3, AGL8, AGL15*, and *AGL32*, was reported to be suppressed by cold stress (Hannah et al., 2005), implying a role for Ca^2+^ signal to regulate cold responses through the MADS proteins, however, whether and how Ca^2+^/CaM regulates MADS box transcription factors remain to be addressed. The expression of *CBF2* is down-regulated in transgenic *Arabidopsis* plants constitutively expressing *AGL15*, in comparison to WT plants (Hill et al., 2008). GT factors are plant-specific transcription factors sharing a conserved trihelix DNA-binding domain that specifically interacts with GT *cis*-elements (Wang et al., 2014). Recently, AtGT2L, a classic member of the GT-2 subfamily, was identified to encode a Ca^2+^-dependent CBP and it is responsive to cold, salt and ABA treatments (Xi et al., 2012). Furthermore, overexpression of *AtGT2L* resulted in elevated expression levels of cold- and salt-specific marker genes *RD29A* and *ERD10*, both in basal and chilling- or salt-treated conditions. These results indicated that Ca^2+^/CaM-binding AtGT2L is involved in plant responses to cold and salt stresses (Xi et al., 2012).

### Salt and Drought Stress

High salinity and drought are the major environmental stresses frequently experienced by plants, and both impose osmotic stress on plant cells. Osmotic stress induces a series of responses at the molecular and cellular levels and a primary event is an increase in the cytosolic Ca^2+^ concentration and subsequent transduction of Ca^2+^ signals that promotes appropriate cellular responses in an effort to mitigate potential damages (Xiong and Zhu, 2002; Zhu, 2002). In addition to the well documented salt-overly-sensitive (SOS) pathway (Chinnusamy et al., 2004; Mahajan et al., 2008), CaM-mediated signaling is also actively involved in plant response to osmotic stress (Bouche et al., 2005). Overexpression of a salt-induced CaM gene from soybean, *GmCaM4*, in *Arabidopsis* confers salt stress tolerance through the up-regulation of DNA-binding activity of a MYB transcription factor MYB2. Interestingly, MYB2 was also reported to interact with CaM in a Ca^2+^-dependent manner and regulate salt and dehydration responsive genes (Abe et al., 2003; Yoo et al., 2005). *AtCML8*, an ortholog of *GmCaM4*, was also found to be induced by salt treatment (Park et al., 2010). Another similar CML protein AtCML9 was found to be involved in osmotic stress tolerance through ABA-mediated pathways (Magnan et al., 2008). *AtCML9* was readily induced by abiotic stress and ABA; knock-out mutant *atcml9* showed a hypersensitive response to ABA during seed germination and seedling growth stages, and exhibited enhanced tolerance to salt and dehydration stresses. Furthermore, expression of several stress and ABA-responsive genes including *RAB18, RD29A*, and *RD20* was altered in *atcml9*. The rice CML gene *OsMSR2* was also suggested to be involved in ABA-mediated salt and drought tolerance (Xu et al., 2011). As the most abundant vacuolar Na^+^-proton exchanger in *Arabidopsis*, Na^+^/H^+^ exchanger 1 (AtNHX1) regulates various cellular activities such as maintaining pH, ion homeostasis, and protein trafficking. Yamaguchi et al. (2005) found that AtCaM15 (also called AtCML18) is localized in the vacuolar lumen and interacts with the C-terminus of AtNHX1. The interaction between AtCaM15 and AtNHX1 is affected by both Ca^2+^ and pH, and the binding of AtCaM15 to AtNHX1 alters the Na^+^/K^+^ selectivity of the exchanger by decreasing its Na^+^/H^+^ exchange speed. The interaction between AtCaM15 and AtNHX1 suggests the presence of Ca^2+^-pH-dependent signaling components in the vacuole, which are involved in mediating plant responses to salt stress. In addition to the above mentioned CaM/CMLs, *CML37, CML38*, and *CML39* are also responsive to various stimuli, including salt, drought, and ABA (Vanderbeld and Snedden, 2007), but whether they are also involved in osmotic stress tolerance remains to be identified.

A few CaMBPs are involved in the signaling pathways triggered by salt, drought or osmotic stresses. Wheat (*Triticum aestivum*) *TaCCaMK* was down-regulated by ABA, salt and PEG treatments, and overexpression of *TaCCaMK* reduces ABA sensitivity of *Arabidopsis*, indicating that TaCCaMK is a negative regulator of ABA-mediated signaling (Yang et al., 2011). *Arabidopsis AtACA4* encoding a CaM-regulated Ca^2+^-ATPase was found to be localized to small vacuoles, which is similar to PMC1, the yeast vacuolar Ca^2+^-ATPase, and AtACA4 confers tolerance against osmotic stresses imposed by high NaCl, KCl, and mannitol, when expressed in the yeast K616 strain lacking Ca^2+^ transporter PMC1 (Geisler et al., 2000). A CaM-regulated Ca^2+^-ATPase gene from soybean, *SCA1*, was found to be induced by salt stress (Chung et al., 2000). Methylglyoxal (MG), a byproduct of carbohydrate and lipid metabolism and a potent mutagenic chemical known to arrest growth, reacts with DNA and protein and increases sister chromatid exchange; and glyoxalase enzymes, including glyoxalase I (gly-I) and glyoxalase II (gly-II), catalyze the detoxification of MG with the involvement of glutathione (GSH; Thornalley, 1990). Glyoxalase I from *Brassica juncea* (BjGly-I) was reported to be a Ca^2+^/CBP, and its enzymatic function is significantly stimulated by Ca^2+^/CaM binding (Deswal and Sopory, 1999). The expression of *BjGly-I* is induced by salt, dehydration and heavy metal stresses; ectopic expression of *BjGly-I* in tobacco conferred remarkable tolerance to exogenous MG and high salt stress (Veena et al., 1999). AtCaMBP25 was identified to be a CaM-binding nuclear protein and is induced by dehydration, low temperature or high salinity. Overexpression of *AtCaMBP25* compromised the tolerance of transgenic plants to osmotic stress, and silencing *AtCaMBP25 via* antisense approach increased plant tolerance to osmotic stress. These results suggested that AtCaMBP25 functions as a negative regulator in plant tolerance to osmotic stress, revealing a connection coupling Ca^2+^ signals to plant responses to osmotic stresses (Perruc et al., 2004).

Ca^2+^/CaM-regulated transcription factors are also involved in plant response to salt and drought stresses. A few *CAMTA* genes from *Arabidopsis* and soybean are up-regulated by salt and dehydration treatments (Yang and Poovaiah, 2002a; Galon et al., 2010; Wang et al., 2015). *Arabidopsis* CAMTA1 is involved in drought stress response (Pandey et al., 2013). Knockout mutant *camta1* was shown to be more sensitive to drought stress, and expression of many drought responsive genes was affected in this mutant. Similar to AtCAMTA1, tomato CAMTA homolog SlSR1L was also positively involved in drought stress tolerance (Li et al., 2014). In addition to regulating salicylic acid (SA)-induced defense response and systemic acquired resistance (Wang et al., 2009; Zhang et al., 2010), AtCBP60g, a CaM-binding transcription factor from *Arabidopsis* was found to positively regulate drought stress response (Wan et al., 2012). Transgenic plants overexpressing *CBP60g* displayed hypersensitivity to ABA and enhanced tolerance to drought stress. AtGTL1 (GT-2 LIKE1), a CaM-binding member of the GTL transcription factor family, was found to be a negative regulator of drought tolerance (Yoo et al., 2010). *AtGTL1* expression was down-regulated by dehydration stress, and loss-of-function mutant *gtl1* showed better survival under drought stress by reducing transpiration, due to lower stomata density on the abaxial surface and higher expression of *SDD1*, which is a negative regulator of stomatal development and is repressed by AtGTL1 (Yoo et al., 2010). Similarly, PtaGTL1 identified from *Populus tremula* × *P. alba* could bind to CaM and regulate water use efficiency and drought tolerance (Weng et al., 2012). Another transcription factor AtABF2/AREB1, which was identified as CBP through protein microarray analysis (Popescu et al., 2007), was also found to be up-regulated by ABA, dehydration, and salinity stresses (Yoshida et al., 2010). Single and multiple mutants of ABF2, 3, and 4 showed varying degrees of reduced survival rate under drought conditions, implying functional redundancy among these three ABFs and Ca^2+^/CaM could regulate drought tolerance through ABF2/AREB1 (Yoshida et al., 2010).

### Heavy Metal Stress

Elevated concentration of both essential (e.g., Cu and Zn) and non-essential (e.g., Cd, Hg, Pb, and Ni) heavy metals in the soil can cause toxicity and inhibit plant growth. It was reported that Ca^2+^/CaM is involved in radish (*Raphanus sativus* L.) responses to Cd^2+^ toxicity during the early phases of seed germination (Rivetta et al., 1997). Ca^2+^ added in the medium could partially reverse the Cd^2+^-induced growth inhibition of the germinating embryo, and this coincides with decreased Cd^2+^ uptake. An equilibrium dialysis study revealed that Cd^2+^ compete with Ca^2+^ for CaM-binding, hence Cd^2+^ could significantly reduce the binding of Ca^2+^/CaM to its target proteins. Apparently, supplementation of Ca^2+^ in the medium counteracts the toxicity of Cd^2+^ by restoring the Ca^2+^-dependent interaction between CaM and its targets during the radish seed germination. A tobacco (*N. tabacum*) cyclic nucleotide gated ion channel (CNGC) called NtCBP4 was identified to be a CBP through protein–protein interaction-based library screening, and shown to be localized to plasma membrane. Transgenic tobacco plants with elevated expression of *NtCBP4* displayed tolerance to Ni^2+^ and hypersensitivity to Pb^2+^, and consistently showed decreased Ni^2+^ and increased Pb^2+^ accumulation, suggesting that NtCBP4 is involved in heavy metal uptake across the plant plasma membrane (Arazi et al., 1999). However, transgenic plants expressing a truncated version of NtCBP4 lacking the C-terminal stretch covering the CaMBD and part of the putative cyclic nucleotide-binding domain showed improved tolerance to Pb^2+^ and lower accumulation of Pb^2+^, and loss-of-function mutation of AtCNGC1, a homolog of NtCBP4 in *Arabidopsis*, also resulted in Pb^2+^ tolerance. These results suggested that CaM-binding is required for the normal function of both AtCNGC1 and NtCBP4 for the transport of heavy metals (Sunkar et al., 2000).

## Conclusion and Perspectives

**FIGURE 1:**
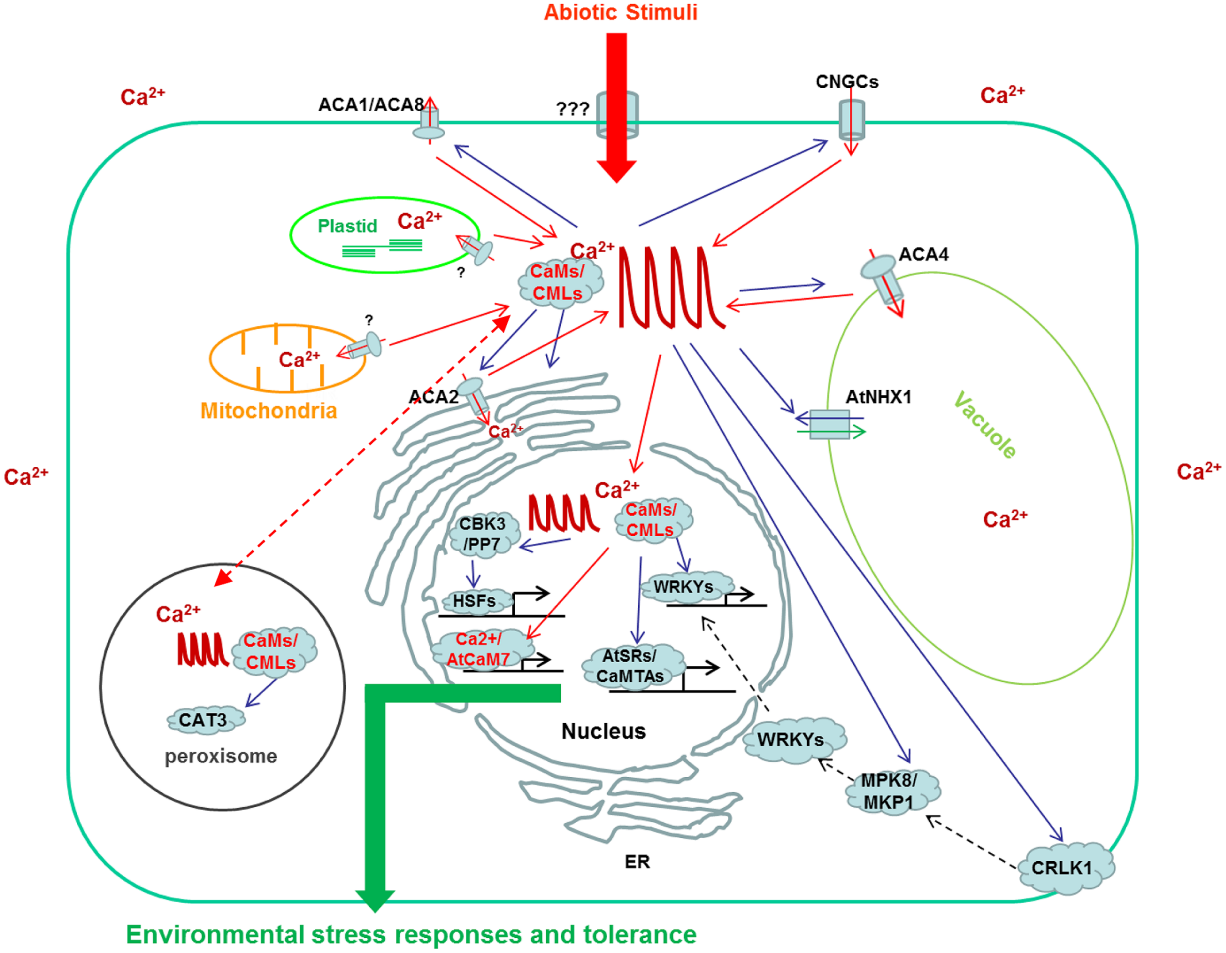
**Schematic representation of Ca^2+^ transients and their modification and interpretation by CaM/CMLs as well as their target proteins in plant cells under abiotic stresses.** This model is not exhaustive and only includes the actions of a limited number of CaM/CMLs and target proteins; CaMs/CMLs/CBPs involved in biotic stresses and Ca^2+^ signal interpretation by other sensors such as CBLs and CDPKs are not included. Actions modifing Ca^2+^ transients or CaM/CMLs are presented by red arrows and actions regulated by Ca^2+^/CaMs or Ca^2+^/CMLs are presented by blue arrows. The dashed arrows imply multiple regulations extended to nucleus.

Ca^2+^ is a critical second messenger coupling diverse stimuli to various physiological responses in plants. CaM, as well as CML, is one of the most extensively studied Ca^2+^ sensors, which mediate interpretation of Ca^2+^ signals in all aspects of plant life, especially in responses to environmental stresses, through interaction with and regulation of various downstream target proteins. **Figure 1** depicts an overview of generation and interpretation of Ca^2+^ signals which are regulated by CaM/CMLs during plant responses to abiotic stresses. One of the most actively regulated class of target proteins are calcium permeable channels, pumps, and antiporters which are actively involved in the generation of intracellular Ca^2+^ transients. This indicates that the preciseness and accuracy of Ca^2+^ signal itself is closely monitored by CaM-mediated regulation. Although more than 50 proteins from different plant species have been identified as CBPs with well-defined CaM-binding properties (**Table 1**), the CaM-mediated regulations of these target proteins are frequently presumptive including SAURs, PCBP, AtBTs, and WRKYIIds (Yang and Poovaiah, 2000a; Reddy et al., 2002; Du and Poovaiah, 2004; Park et al., 2005). Only a few cases of CaM-mediated regulation *in planta* are supported with empirical evidences, such as GAD, CCaMK, AtCAT3, MLO, DWF1, AtSRs/CaMTAs, CRLK1, and CBP60g (Snedden et al., 1996; Kim et al., 2002; Yang and Poovaiah, 2002b; Du and Poovaiah, 2005; Gleason et al., 2006; Tirichine et al., 2006; Du et al., 2009; Wang et al., 2009; Yang et al., 2010b). Hence, more emphasis should be placed on studying the CaM-mediated regulation of target proteins to further improve our understanding of CaM-mediated signaling. Currently, most of the CBPs are targets of canonic CaMs which count for only 10% of the CaM/CML family. The targets of most of the CMLs are not reported yet, let alone the CML-mediated regulation of downstream targets and associated signal transduction. Identification of novel target proteins of CaMs and CMLs especially those interact with CMLs deserve special attentions. Environmental cues are known to trigger stimulus specific Ca^2+^ transients. In an effort to explain how the simple Ca^2+^ ion could act as a messenger to couple various environmental stimuli to appropriate physiological responses with astonishing accuracy, Webb et al. (1996) proposed the theory of “Ca^2+^ signature” which hypothesized that stimulus-triggered increases in intracellular Ca^2+^ concentration vary in terms of duration, frequency, amplitude, and spatial distribution, and these carry specific information when they are interpreted into different physiological responses. An obvious support for this hypothesis is that the different Ca^2+^ spikes triggered by Nod factor from rhizobia and Myc factor from arbuscular mycorrhizal fungi could be interpreted through the action of the same Ca^2+^, Ca^2+^/CaM dependent protein kinase, CCaMK, into different physiological responses to support the establishment of root nodulation symbiosis or arbuscular mycorrhization (Kosuta et al., 2008). Although exciting progress on how Ca^2+^ signals are interpreted into various physiological responses has been made in the last decade, what we know so far may be very limited in scope when one considers the complicated Ca^2+^ signaling network. Many issues such as specificity, preference and flexibility of interaction between various CaM/CMLs and target proteins *in planta* are barely understood. The dynamics of Ca^2+^/CaM mediated regulation, the mechanistic details by which a particular effector detects a difference in Ca^2+^ signature and initiates distinct signaling pathways, are basically unknown. Progress in addressing these issues will help in understanding the most amazing properties, the versatility, efficiency and accuracy of Ca^2+^-mediated signaling in plant responses to environmental stresses.

## Conflict of Interest Statement

The authors declare that the research was conducted in the absence of any commercial or financial relationships that could be construed as a potential conflict of interest.
